# Noninvasive Immunotyping and Immunotherapy Monitoring of Lung Cancers via Nuclear Imaging of LAG‐3 and PD‐L1

**DOI:** 10.1002/advs.202404231

**Published:** 2024-11-08

**Authors:** Lishu Zhao, Jianxian Ge, Ruru Zhang, Hao Wang, Xinyue Liu, Kandi Xu, Yujin Liu, Wencheng Zhao, Wengang Zhang, Li Ye, Zhimin Chen, Jianfeng Zeng, Yayi He, Mingyuan Gao

**Affiliations:** ^1^ Department of Medical Oncology Shanghai Pulmonary Hospital Tongji University Medical School Cancer Institute School of Medicine Tongji University No 507 Zhengmin Road Shanghai 200433 China; ^2^ Center for Molecular Imaging and Nuclear Medicine State Key Laboratory of Radiation Medicine and Protection School for Radiological and Interdisciplinary Sciences (RAD‐X) Collaborative Innovation Center of Radiation Medicine of Jiangsu Higher Education Institutions Soochow University Suzhou 215123 China

**Keywords:** lung cancer, nuclear imaging, SPECT, LAG‐3, PD‐L1, immunotherapy, immunotype

## Abstract

Immunotherapy has significantly improved cancer patient survival, while its efficacy remains limited due to the reliance on a single marker like PD‐L1 as well as its spatiotemporal heterogeneity. To address this issue, combining lymphocyte activation gene‐3 (LAG‐3) with PD‐L1 is proposed for identifying immunotypes and monitoring immunotherapy through nuclear imaging. In short, ^99m^Tc‐HYNIC‐αLAG‐3 and ^99m^Tc‐HYNIC‐αPD‐L1 probes are synthesized using anti‐human LAG‐3 and PD‐L1 antibodies, respectively. With high radiochemical purity and in vitro stability, these probes are confirmed to specifically bind to LAG‐3 or PD‐L1 in LAG3^+^ A549, LAG3^−^ A549, and H1975 cells. SPECT/CT imaging of both probes showed specific in vivo tumor uptake in multiple lung cancer models, with significant linear correlation with ex vivo tumor uptake and immunohistochemical expression levels of LAG‐3/PD‐L1. Based on this, dual‐index imaging was performed to simultaneously quantify LAG‐3 and PD‐L1. SPECT/CT imaging of ^99m^Tc‐HYNIC‐αLAG‐3 and ^125^I‐αPD‐L1 successfully distinguished four immunotypes. In addition, SPECT/CT imaging revealed LAG‐3 upregulation in LLC‐bearing LAG‐3 humanized mice resistant to immunotherapy. In conclusion, this study demonstrates the feasibility of nuclear imaging of LAG‐3 and PD‐L1 for both noninvasive immunotyping and immunotherapy monitoring, thus offering novel perspectives on forecasting immunotherapy response, uncovering resistance mechanism, and optimizing combination treatment regimens.

## Graphical abstract

SPECT/CT imaging of lung cancers with ^99m^Tc‐HYNIC‐αLAG‐3 and ^99m^Tc‐HYNIC‐αPD‐L1 probes represents an effective method for noninvasively visualizing LAG‐3 and PD‐L1, and immunotherapy resistance monitoring. Additionally, the pioneering simultaneous imaging of LAG‐3 and PD‐L1 allows for distinguishing various lung cancer immunotypes, which is very valuable for improving the immunotherapeutic efficacy by properly selecting immune checkpoint inhibitors as well as their optimal intervention timing.

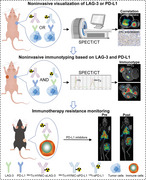

## 1 Introduction

Lung cancers exhibit the highest mortality rates worldwide.^[^ ^1^ ^]^ Immunotherapy represented by programmed cell death 1/programmed cell death ligand 1 (PD‐1/PD‐L1) inhibitors has revolutionized the treatment regimens for advanced lung cancers.^[^ ^1^, ^2^ ^]^ However, the overall response rate remains far from satisfactory, primarily attributed to the exclusive reliance on PD‐L1 as a solitary immunotherapy marker in clinical settings, as well as its complex spatiotemporal heterogeneity.^[^ ^2^, ^3^ ^]^

The expression level of PD‐L1 on tumor cells, determined through immunohistochemistry (IHC), serves as a cornerstone for choosing immune monotherapy or combination therapy in clinical practice. However, in various cancers, especially in lung cancers, multiple stimulatory and inhibitory molecules have been found to be co‐expressed with PD‐1/PD‐L1, including lymphocyte activation gene 3 (LAG‐3), T cell immunoglobulin domain and mucin domain‐3, and cytotoxic T lymphocyte antigen 4 (CTLA‐4).^[^ ^4^ ^]^ Integrating additional immune checkpoints (ICPs) with PD‐L1 to enhance the prediction accuracy and treatment efficacy is of great clinical significance.

LAG‐3, a third‐generation ICP following PD‐1 and CTLA‐4, has attracted extensive attention, particularly after the U.S. Food and Drug Administration has approved the first LAG‐3‐targeting drug Relatlimab for its superior efficacy when used in combination with PD‐1 inhibitors compared to anti‐PD‐1 monotherapy in melanoma patients.^[^ ^5^ ^]^ Previous studies have shown that 25–50% of patients with untreated non‐small cell lung cancer (NSCLC) are positive for LAG‐3 on tumor‐infiltrating lymphocytes (TILs),^[^ ^4^, ^6^ ^]^ highlighting its potential therapeutic significance. Typically, LAG‐3 predominantly expressed on activated or exhausted immune cells promotes the immunosuppressive function of regulatory T (Treg) cells and hampers the cytotoxic effects of CD8^+^ T cells.^[^ ^7^ ^]^ The expression level of LAG‐3 was also reported to be associated with both primary and adaptive resistance to PD‐1/PD‐L1 inhibition.^[^ ^8^ ^]^ Moreover, lung cancer patients who exhibited low LAG‐3 but high PD‐L1 levels were found to have remarkably prolonged progression‐free survival (PFS) in response to PD‐1 blockade therapy, in contrast to those with high LAG‐3 but low PD‐L1 levels.^[^ ^4a^ ^]^ Additionally, LAG‐3 combined with PD‐1 blockades exhibited prolonged PFS in melanoma patients with LAG‐3 expression ≥ 1%.^[^ ^5^ ^]^ Therefore, evaluating LAG‐3 expression, in conjunction with PD‐L1 levels, may not only be valuable for predicting PD‐1/PD‐L1 inhibitor efficacy but also for tailoring the combined treatment regimens.

Traditional IHC tests, commonly used in clinics to assess efficacy markers, are facing limitations in fully illustrating the temporal and spatial heterogeneity of the tumor microenvironment. To overcome this challenge, versatile nuclear medicine imaging probes, typically comprised of targeting ligands, linkers, chelators, and radionuclides, have been extensively explored for tumor imaging. For instance, positron emission tomography (PET) imaging using ^89^Zr‐radiolabeled PD‐L1 antibodies has enabled noninvasive quantification of PD‐L1.^[^ ^3^, ^9^ ^]^ Similarly, single photon emission computed tomography (SPECT) imaging of ^99m^Tc‐radiolabeled LAG‐3‐targeting nanobodies enables dynamic visualization of LAG‐3 on TILs.^[^ ^10^ ^]^ Despite these significant advancements, the correlation between tracer tumor uptake and LAG‐3 expression level determined by IHC remains incompletely illustrated. Furthermore, there is a lack of preclinical or clinical investigations regarding simultaneous nuclear imaging of PD‐L1 and LAG‐3.

In this study, we performed SPECT/CT imaging of ^99m^Tc‐HYNIC‐αLAG‐3 and ^99m^Tc‐HYNIC‐αPD‐L1 to disclose the correlation between tracer tumor uptake and the expression levels of LAG‐3/PD‐L1. Considering the efficacy prediction value of PD‐L1 and LAG‐3 as well as the resistance monitoring potential of LAG‐3, dual index imaging of ^99m^Tc‐HYNIC‐αLAG‐3 and ^125^I‐αPD‐L1 was conducted to enable simultaneous visualization of LAG‐3 and PD‐L1, which was the first time to achieve noninvasive immunotyping in lung cancers. The current strategy also allowed surveillance on LAG‐3 expression in lung cancers during immunotherapy. In brief, the current study has important implications for improving the predictive precision of immunotherapy efficacy and promoting the optimization of immune combination regimens, apart from the substantial potential for clinical translation.

## 2 Results

### 2.1 Preparation of ^99m^Tc‐HYNIC‐αLAG‐3 and ^99m^Tc‐HYNIC‐αPD‐L1 Probes

**Figure 1:**
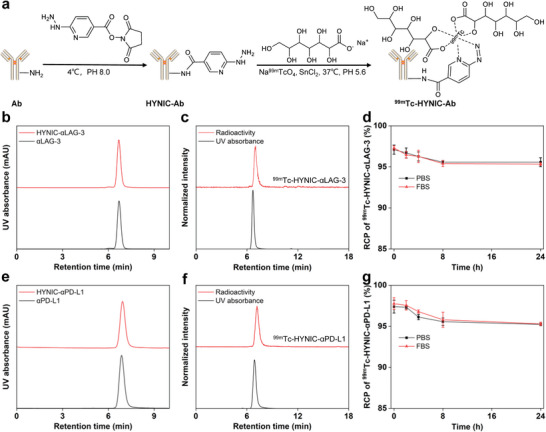
Preparation of ^99m^Tc‐HYNIC‐αLAG‐3 and ^99m^Tc‐HYNIC‐αPD‐L1 probes. a) The synthetic route of ^99m^Tc‐radiolabeled probes. b) Radio‐HPLC chromatograms revealed R_t_ of 6.7 min for αLAG‐3, HYNIC‐αLAG‐3. c) For ^99m^Tc‐HYNIC‐αLAG‐3, the UV absorbance at 280 nm showed R_t_ of 6.7 min, while the radioactivity was noted at 7.0 min. d) In vitro stability of ^99m^Tc‐HYNIC‐αLAG‐3. e) HPLC chromatograms of αPD‐L1 and HYNIC‐αPD‐L1 with R_t_ of 6.9 min. f) Radio‐HPLC chromatograms of ^99m^Tc‐HYNIC‐αPD‐L1 revealed retention time of 6.9 min for UV absorbance at 280 nm and 7.2 min for radioactivity, respectively. g) In vitro stability ^99m^Tc‐HYNIC‐αPD‐L1. For insets d and g, data are presented as mean ± SD, *n* = 3. Ab: antibody; RCP: radiochemical purity; HPLC: high‐performance liquid chromatography; R_t_: retention time.

As depicted in **Figure** **1**a, antibodies were conjugated with succinimidyl‐hynic hydrochloride (HYNIC‐NHS) and then labeled with ^99m^Tc to obtain imaging probes targeting LAG‐3 and PD‐L1. High‐performance liquid chromatography (HPLC) chromatograms, as delineated in Figure 1b, revealed a chemical purity of over 99% for both αLAG‐3 and HYNIC‐αLAG‐3. ^99m^Tc‐HYNIC‐αLAG‐3 achieved radiolabeling yields of 57.1 ± 3.0% and ^99m^Tc‐radiocolloid formation rate < 5%, with radiochemical purity (RCP) exceeding 95%, as confirmed by radio‐HPLC (Figure 1c; Figure S1, Supporting Information; *n* = 3). Moreover, the RCP of ^99m^Tc‐HYNIC‐αLAG‐3 was verified over 24 h, showing in vitro stability of 95.6 ± 0.6% in PBS and 95.3 ± 0.2% in FBS, and in vivo stability of 95.0 ± 0.5% (Figure 1d; Figure S2, Supporting Information; *n* = 3). Similarly, αPD‐L1 and HYNIC‐αPD‐L1 exhibited a chemical purity surpassing 99%, with ^99m^Tc‐HYNIC‐αPD‐L1 showing radiolabeling yields of 50.5 ± 5.2% and ^99m^Tc‐radiocolloid percentage < 5% (Figure 1e; Figure S3, Supporting Information). ^99m^Tc‐HYNIC‐αPD‐L1 remained stable over 24 h, demonstrating 95.2 ± 0.2% stability in PBS, 95.3 ± 0.2% in FBS, and 94.3 ± 0.8% in vivo (Figure 1f,g; Figure S4, Supporting Information; *n* = 3). The findings indicate that both ^99m^Tc‐HYNIC‐αLAG‐3 and ^99m^Tc‐HYNIC‐αPD‐L1 have high RCP and stability under physiological conditions within 24 h.

### 2.2 Binding Capacity Evaluation of ^99m^Tc‐HYNIC‐αLAG‐3 and ^99m^Tc‐HYNIC‐αPD‐L1 In Vitro

**Figure 2:**
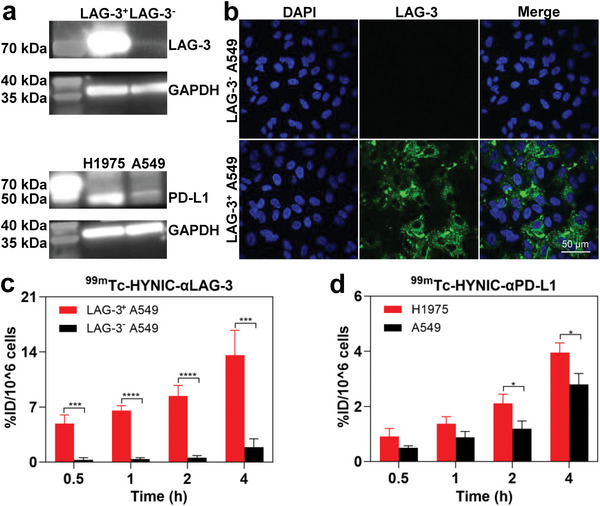
Binding capacity evaluation of ^99m^Tc‐HYNIC‐αLAG‐3 and ^99m^Tc‐HYNIC‐αPD‐L1 in vitro. a) WB analyses of LAG‐3 and PD‐L1 expression. b) Immunofluorescence of LAG‐3. The cell binding rate of ^99m^Tc‐HYNIC‐αLAG‐3 c) and ^99m^Tc‐HYNIC‐αPD‐L1 d). For insets c and d, data are presented as mean ± SD, *n* = 3. ^*^ *p* < 0.05, ^***^ *p* < 0.001, ^****^ *p* < 0.0001; by unpaired *t*‐tests.

In vitro analysis revealed a differential expression of LAG‐3 protein, with LAG‐3 transfected A549 (LAG‐3^+^ A549) exhibiting expression, contrary to wild‐type A549 cells (**Figure** **2**a,b). PD‐L1 was highly expressed on H1975 cells compared to A549 cells (Figure 2a). Within 4 h, the binding rates of ^99m^Tc‐HYNIC‐αLAG‐3 to LAG‐3^+^ A549 cells gradually increased, exhibiting significantly higher rates than LAG‐3^−^ A549 cells, particularly at 4 h (13.6 ± 3.2% vs 1.9 ± 1.1%, *p* < 0.001; *n* = 3; Figure 2c). Regarding ^99m^Tc‐HYNIC‐αPD‐L1, H1975 presented a greater cell binding efficiency than A549 cells at 2 and 4 h (2 h: 2.1 ± 0.3% vs 1.2 ± 0.3%; 4 h: 4.0 ± 0.4% vs 2.8 ± 0.4%, both *p* < 0.05; *n* = 3; Figure 2d). These results indicate that both probes can bind specifically to the corresponding protein targets.

### 2.3 SPECT/CT Imaging of ^99m^Tc‐HYNIC‐αLAG‐3 and ^99m^Tc‐HYNIC‐αPD‐L1 In Vivo

**Figure 3:**
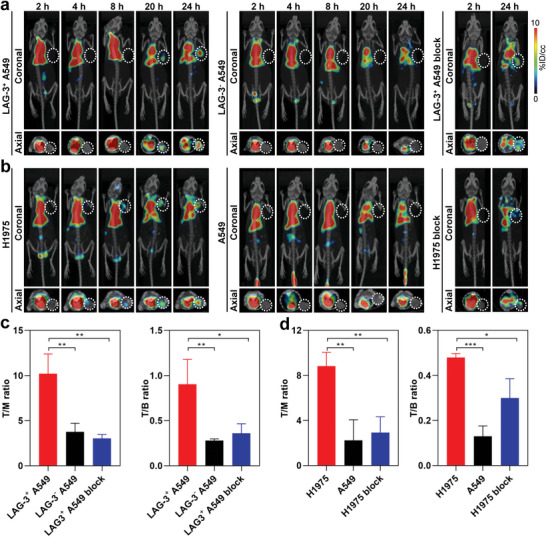
SPECT/CT imaging of ^99m^Tc‐HYNIC‐αLAG‐3 and ^99m^Tc‐HYNIC‐αPD‐L1 in lung cancer models. Representative coronal and axial SPECT/CT images of ^99m^Tc‐HYNIC‐αLAG‐3 a) and ^99m^Tc‐HYNIC‐αPD‐L1 b). T/M and T/B ratio of ^99m^Tc‐HYNIC‐αLAG‐3 c) and ^99m^Tc‐HYNIC‐αPD‐L1 d). White dotted circles indicate tumors. For insets c and d, data are presented as mean ± SD, *n* = 3. ^*^ *p* < 0.05, ^**^ *p* < 0.01, ^***^ *p* < 0.001; by unpaired *t*‐tests. T/M: tumor to muscle; T/B: tumor to blood.

SPECT/CT imaging of ^99m^Tc‐HYNIC‐αLAG‐3 was performed in LAG‐3^+^ A549 and LAG‐3^−^ A549 mice models (*n* = 3). Representative coronal and axial images captured at 2, 4, 8, 20, and 24 h post‐injection revealed that LAG‐3^+^ A549 mice presented noticeable signal from tumor site commencing from 20 h post‐injection, peaking at 24 h, in contrast to LAG‐3^−^ A549 mice that showed no noticeable signal at the given timepoint (**Figure** **3**a). Moreover, tumor uptake can be partially blocked by unlabeled αLAG‐3, demonstrating its specific binding capacity. Quantitatively, the tumor‐to‐muscle (T/M) and tumor‐to‐blood (T/B) ratios in LAG‐3^+^ A549 mice were statistically higher than those in LAG‐3^−^ A549 mice (T/M: 10.2 ± 2.2 vs 3.8 ± 0.9, *p* < 0.01; T/B: 0.9 ± 0.3 vs 0.3 ± 0.02, *p* < 0.01) and blocked LAG‐3^+^ A549 mice (T/M: 10.2 ± 2.2 vs 3.0 ± 0.4, *p* < 0.01; T/B: 0.9 ± 0.3 vs 0.4 ± 0.1, *p* < 0.05) (Figure 3c). Likewise, SPECT/CT imaging of ^99m^Tc‐HYNIC‐αPD‐L1 indicated that H1975 mice exhibited significantly greater tumor signal compared to A549 mice (T/M: 8.8 ± 1.2 vs 2.2 ± 1.8, *p* < 0.01; T/B: 0.5 ± 0.02 vs 0.1 ± 0.05, *p* < 0.01; *n* = 3) and blocked H1975 mice (T/M: 8.8 ± 1.2 vs 2.9 ± 1.4, *p* < 0.001; T/B: 0.5 ± 0.02 vs 0.3 ± 0.09, *p* < 0.05; *n* = 3) (Figure 3b,d). The results demonstrate that both probes can well bind with their corresponding targets, showing excellent binding specificity in lung cancer models.

Post 24‐h scanning, mice administered with ^99m^Tc‐radiolabeled probes were euthanized to delineate the *ex vivo* biodistribution. The radioactivity distribution of ^99m^Tc‐HYNIC‐αLAG‐3 predominantly encompassed blood, liver, spleen, lung, and kidney, with no notable variance among LAG‐3^+^ A549, LAG‐3^−^ A549, and blocked LAG‐3^+^ A549 mice (non‐tumor tissues: *p* > 0.05, Figure S5a, Supporting Information). A comparable biodistribution profile was also observed for ^99m^Tc‐HYNIC‐αPD‐L1 as well (Figure S5b, Supporting Information).

### 2.4 Correlation between Tumor Expressions of LAG‐3/PD‐L1 and Tracer Tumor Uptake In Vivo

**Figure 4:**
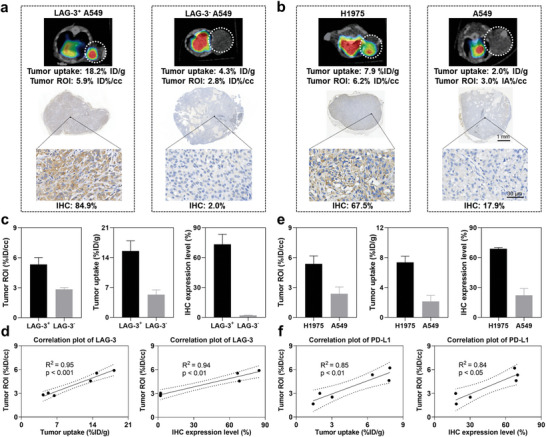
Correlation between tumor expressions of LAG‐3/PD‐L1 and tracer tumor uptake in vivo. Higher tumor uptake of ^99m^Tc‐radiolabeled probes corresponds to increased expression levels of LAG‐3 a) or PD‐L1 b), as evidenced by IHC. Tumor uptake quantified by ROI analyses, tumor uptake determined by ex vivo γ‐counting, IHC expression levels, and correlation plots of tumor uptake in ROI (y‐axis) with ex vivo tumor uptake (x‐axis) or IHC expression levels (x‐axis) for ^99m^Tc‐HYNIC‐αLAG‐3 c,d) and ^99m^Tc‐HYNIC‐αPD‐L1 e,f). For insets c and e, data are presented as mean ± SD, *n* = 3. For insets d and f, *p* value was calculated using Pearson correlation analyses. White dotted circles indicate tumors. ROI: region of interest; IHC: immunohistochemistry.

After observing increased tumor signals of ^99m^Tc‐radiolabeled probes in tumors with elevated expression of LAG‐3 or PD‐L1 (**Figure** **4**a,b), we further quantified the correlations among in vivo tumor uptake obtained by region of interest (ROI) analysis, tumor uptake determined through ex vivo γ‐counting, and IHC expression levels. Regarding ^99m^Tc‐HYNIC‐αLAG‐3, LAG‐3^+^ A549 tumors presented an average *ex vivo* tumor uptake of 15.5 ± 2.4 %ID/g, in vivo tumor uptake of 5.3 ± 0.7 %ID/cc, and LAG‐3 expression level of 73.3 ± 10.0%, while these values became 5.3 ± 1.1 %ID/g, 2.8 ± 0.2 %ID/cc, and 2.1 ± 0.2%, respectively, for LAG‐3^−^ A549 tumors (Figure 4c; *n* = 3). Significant correlations were observed between in vivo tumor uptake and *ex vivo* tumor uptake (R^2^ = 0.95, *p* < 0.001) as well as LAG‐3 expression level (R^2^ = 0.94, *p* < 0.01) in the Pearson correlation analyses (Figure 4d).

Upon injection of ^99m^Tc‐HYNIC‐αPD‐L1, H1975 tumors provided an average *ex vivo* tumor uptake of 7.1 ± 0.7 %ID/g and in vivo tumor uptake of 5.4 ± 0.8 %ID/cc, significantly exceeding the corresponding values of A549 tumors, which were 2.1 ± 0.8 %ID/g (*p* < 0.01) and 2.4 ± 0.7 %ID/cc (*p* < 0.01), respectively (Figure 4e). Correspondingly, PD‐L1 levels were higher in H1975 tumors vs A549 tumors (68.8 ± 1.3% vs 22.1 ± 6.9%, *p* < 0.001) (Figure 4e). Similar correlations were observed between in vivo tumor uptake and *ex vivo* tumor uptake (R^2^ = 0.85, *p* < 0.01), as well as the expression level of PD‐L1 (R^2^ = 0.84, *p* < 0.05) (Figure 4f). In short, tracer tumor uptake of ^99m^Tc‐HYNIC‐αLAG‐3 and ^99m^Tc‐HYNIC‐αPD‐L1 on SPECT/CT correlates robustly and linearly with the expression levels of the corresponding targets, thus underscoring the potential of these probes for quantitative evaluation of the corresponding biomarkers in vivo.

### 2.5 Noninvasive Immunotyping through SPECT/CT Imaging of LAG‐3 and PD‐L1

**Figure 5:**
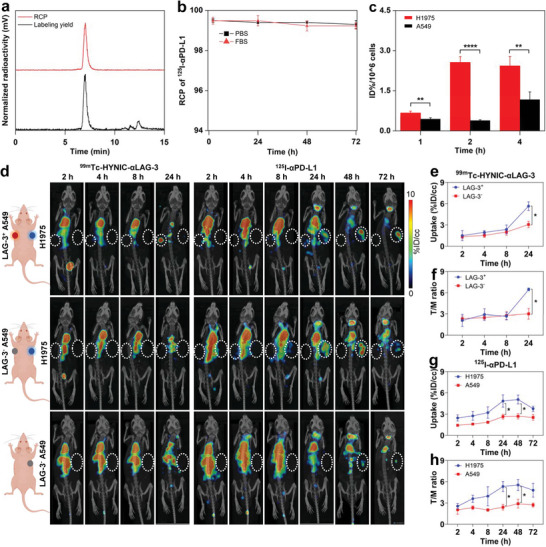
Noninvasive immunotyping through SPECT/CT imaging of LAG‐3 and PD‐L1. a) The labeling yield and RCP of ^125^I‐αPD‐L1. b) In vitro stability of ^125^I‐αPD‐L1 in PBS and 10% FBS (*n* = 3). c) The cell binding rate of ^125^I‐αPD‐L1 (*n* = 3). d) Synchronous SPECT/CT imaging of ^99m^Tc‐HYNIC‐αLAG‐3 and ^125^I‐αPD‐L1 in lung cancer models with three immunotypes. Tumor uptake in ROIs and T/M ratio of ^99m^Tc‐HYNIC‐αLAG‐3 (e‐f; LAG‐3^+^ A549 [*n* = 2] vs LAG‐3^−^ A549/H1975 [*n* = 5]) and ^125^I‐αPD‐L1 (g,h; PD‐L1^high^ H1975 [*n* = 3] vs PD‐L1^low^ A549 [*n* = 4]). For insets b‐c and e‐h, data are presented as mean ± SD. ^*^ *p* < 0.05, ^**^ *p* < 0.01, ^****^ *p* < 0.0001; by unpaired t tests. White dotted circles indicate tumors. RCP: radiochemical purity; ROI: region of interest; T/M: tumor to muscle.

Due to the distinct energy spectra of ^99m^Tc and ^125^I, as shown in Figure S6 (Supporting Information), ^125^I was also adopted to label anti‐human PD‐L1 antibodies for simultaneously visualizing PD‐L1 and LAG‐3, in combination with ^99m^Tc‐HYNIC‐αLAG‐3. ^125^I‐αPD‐L1 showed 92.9 ± 7.0% labeling yields and high stability: 99.2 ± 0.2% in PBS, 99.1 ± 0.2% in FBS, and 97.5 ± 1.1% in vivo over 72 h (**Figure** **5**a,b; Figure S7, Supporting Information; *n* = 3). Furthermore, the cell binding efficiencies of ^125^I‐αPD‐L1 to H1975 cells at 1, 2, and 4 h were significantly higher than those to A549 cells, corroborating the binding specificity of the resulting probe (1 h: 0.7 ± 0.07% vs 0.4 ± 0.05%, *p* < 0.01; 2 h: 2.6 ± 0.2% vs 0.4 ± 0.03%, *p* < 0.0001; 4 h: 2.4 ± 0.4% vs 1.2 ± 0.3%, *p* < 0.01; *n* = 3; Figure 5c).

Subsequent to the co‐injection of ^99m^Tc‐HYNIC‐αLAG‐3 and ^125^I‐αPD‐L1, tumor‐bearing mice with varying immunotypes exhibited diverse imaging signals, as illustrated in Figure 5d. With respect to LAG‐3^+^PD‐L1^high^ immunotyping, it can be observed that LAG‐3^+^ A549 tumor presented discernible tumor uptake of ^99m^Tc‐HYNIC‐αLAG‐3 at 24 h, and PD‐L1‐high H1975 tumors also displayed a significantly enhanced signal of ^125^I‐αPD‐L1 at 24 h, with signal peak occurring at 48 h. For LAG‐3^−^PD‐L1^high^ immunotyping, only PD‐L1‐high H1975 tumors displayed significant uptake of ^125^I‐αPD‐L1 at 24 and 28 h, while others exhibited minimal signal intensity. Finally, LAG‐3^−^PD‐L1^low^ A549 tumors did not show any noticeable signal from any probe.

The quantification analysis revealed that LAG‐3^+^ A549 tumors possessed an apparently higher tumor signal than LAG‐3^−^ A549 or H1975 tumors at 24 h post‐injection, e.g., 5.7 ± 0.9 vs 3.1 ± 1.0 %ID/cc (ROI; *p* < 0.05) and 6.5 ± 0.3 vs 3.0 ± 1.7 (T/M ratio; *p* < 0.05) (Figure 5e,f). Compared with A549 tumors, H1975 tumors exhibited significantly enhanced signal and T/M ratio of ^125^I‐αPD‐L1 at both 24 h (4.8 ± 1.5 vs 2.7 ± 0.7 %ID/cc, *p* < 0.05; 5.3 ± 1.2 vs 2.4 ± 0.8, *p* < 0.05) and 48 h (5.1 ± 1.1 vs 2.7 ± 0.8 %ID/cc, *p* < 0.05; 5.5 ± 1.4 vs 2.9 ± 1.2, *p* < 0.05) (Figure 5g,h). The biodistribution pattern of ^125^I‐αPD‐L1 showed that thyroid and heart had prominent radioactivity accumulation (Figure S8, Supporting Information). Collectively, these results demonstrate that the combination of ^99m^Tc‐HYNIC‐αLAG‐3 and ^125^I‐αPD‐L1 offers an effective approach for noninvasive immunotyping in lung cancers.

### 2.6 Monitoring LAG‐3 Upregulation in LLC Models Resistant to Immunotherapy

**Figure 6:**
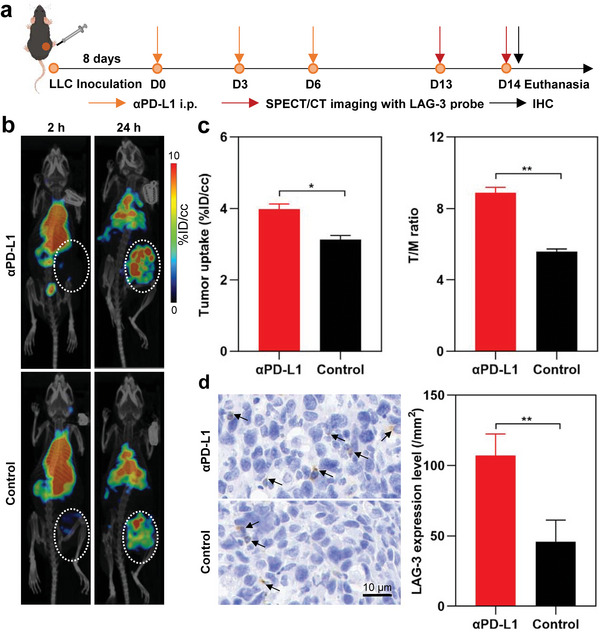
In vivo detecting the upregulation of LAG‐3 in LLC‐bearing LAG‐3 humanized mice resistant to immunotherapy. a) Timeline of immunotherapy and SPECT/CT imaging in LLC‐bearing LAG‐3 humanized mice. b) Representative SPECT/CT images of ^99m^Tc‐HYNIC‐αLAG‐3 at 2 and 24 h after probe injection. c) Tumor uptake and T/M ratio at 24 h after probe injection. d) LAG‐3 expression on IHC between the immunotherapy group and the control group. For insets c and d, data are presented as mean ± SD, *n* = 3. ^*^ *p* < 0.05, ^**^ *p* < 0.01; by unpaired *t*‐tests. White dotted circles indicate tumors. T/M: tumor to muscle; IHC: immunohistochemistry.

As described by the protocol shown in **Figure** **6**a, the ability of ^99m^Tc‐HYNIC‐αLAG‐3 for monitoring the expression of LAG‐3 after PD‐L1 blockade therapy was evaluated in LAG‐3 humanized (hLAG‐3) mice bearing Lewis lung cancer (LLC) that was recognized to be “cold” tumors. After fourteen days following intraperitoneal injection of αPD‐L1, all mice demonstrated resistant to immunotherapy (*p* > 0.05; Figure S9, Supporting Information). SPECT/CT imaging of LLC‐bearing hLAG‐3 mice revealed that those receiving immunotherapy exhibited statistically significant increases in both tumor uptake (4.0 ± 0.2 vs 3.1 ± 0.1 %ID/cc, *p* < 0.05) and T/M ratio (8.9 ± 0.3 vs 5.6 ± 0.1, *p* < 0.05) at 24 h post‐injection of ^99m^Tc‐HYNIC‐αLAG‐3 compared to the control group (Figure 6b,c). Moreover, LAG‐3 upregulation after immunotherapy was confirmed by IHC (Figure 6d). Additionally, the ex vivo biodistribution profiles were comparable between the two groups (Figure S10, Supporting Information). These findings indicate that LAG‐3 SPECT imaging may be potentially useful for noninvasively monitoring the resistance of immunotherapy.

### 2.7 Biological Safety Evaluation

**Figure 7:**
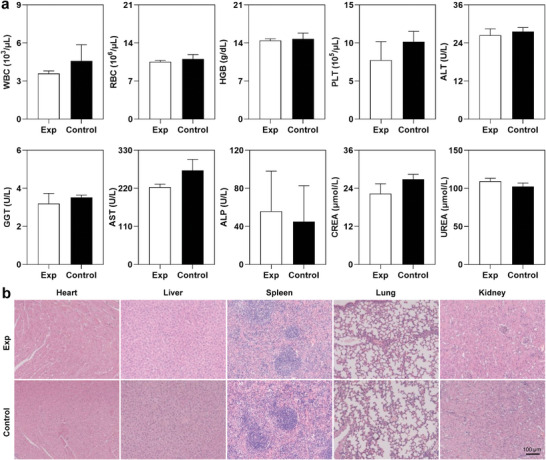
Biological safety evaluation. a) The comparison of blood routine test, liver function, and kidney function between experiment group and control group. Data are presented as mean ± SD, *n* = 3; no significance (*p* > 0.05) by unpaired *t*‐tests. b) HE staining of heart, liver, spleen, lung, and kidney tissues between experiment group and control group. WBC: white blood cells; RBC: red blood cell; PLT: platelet; HGB: hemoglobin; ALT: alanine transaminase; AST: aspartate aminotransferas; ALP: alkaline phosphatas; GGT: gamma‐glutamyl transpeptidase; CREA: creatinine; UREA: urea nitrogen; HE: hematoxylin‐eosin.

In order to evaluate the biological safety of dual‐index imaging using ^99m^Tc and ^125^I, we performed hematological examinations and hematoxylin‐eosin (HE) staining of main organs of tumor‐bearing mice receiving intravenous injection of the imaging probes. The experimental group presented results on nine common blood routine indexes very comparable to those of the control group, as shown in **Figures** **7**a and S11 (Supporting Information). Post‐imaging assessments indicated that liver and kidney functions remained unaltered (Figure 7a). Additionally, HE staining of main organs revealed no discernible morphological changes (Figure 7b). These results suggest that synchronous nuclear imaging with ^99m^Tc and ^125^I does not raise noticeable safety concerns.

## 3 Discussion

Accurately forecasting the potential beneficiary population of immunotherapy has emerged as a crucial issue in the realm of clinical cancer therapy. Excavating and incorporating novel efficacy markers, along with comprehensively elaborating on spatiotemporal heterogeneity, may shed light on selecting the individuals who would benefit from immunotherapy. In comparison with single marker, combining LAG‐3 with PD‐L1 demonstrates superior predictive accuracy for the efficacy of immunotherapy^[^ ^4a^ ^]^ and better prognosis.^[^ ^6^ ^]^ However, simultaneous quantification of LAG‐3 and PD‐L1 has not been achieved in vivo. Herein, we have successfully synthesized ^99m^Tc‐radiolabeled probes targeting LAG‐3 and PD‐L1. With SPECT/CT imaging, we observed strong correlation between tumor SPECT signal and the expression level of the corresponding targets. On this basis, ^125^I‐αPD‐L1 was introduced to facilitate the first‐ever simultaneous imaging of the dual immunotherapy markers LAG‐3 and PD‐L1. Moreover, it was found that LAG‐3 was upregulated after anti‐PD‐L1 therapy resistance, thereby introducing a new way to monitor efficacy markers and immunotherapy resistance as well as overcome the challenges posed by tumor heterogeneity. Thus, these findings offer a novel approach that applies nuclear molecular imaging in assisting with predicting efficacy and selecting either anti‐PD‐1 monotherapy or combined therapy with anti‐LAG‐3 inhibitors.

As for single marker imaging, ^99m^Tc‐HYNIC‐αLAG‐3 and ^99m^Tc‐HYNIC‐αPD‐L1 probes have strong stability and notable affinity for LAG‐3 and PD‐L1 both in vitro and in vivo, respectively. Ex vivo tracer tumor uptake higher than in vivo tumor uptake may be attributed to discrepancies in ROI delineation. PD‐L1, being widely used as an immunotherapy marker in clinics, has prompted the development of numerous nuclear imaging probes for therapeutic efficacy prediction in both preclinical and clinical contexts.^[^ ^11^ ^]^ In a landmark study by Heskamp et al.^[^ ^12^ ^]^ in 2015, ^111^In‐labeled αPD‐L1 was employed for imaging PD‐L1 in tumor models. This was followed by a first‐in‐human clinical trial in which PET imaging with ^89^Zr‐radiolabeled atezolizumab (αPD‐L1) was performed in 22 cancer patients. Notably, the results showed that the tumor PET signal was a more reliable predictor of PD‐L1 inhibitor efficacy than PD‐L1 levels determined through IHC or RNA‐sequencing.^[^ ^3^ ^]^ Long half‐life radioisotopes such as ^89^Zr exhibit a more favorable compatibility with antibodies but also bring longer imaging time window. In our study, both ^99m^Tc‐HYNIC‐αPD‐L1 and ^125^I‐αPD‐L1 can detect PD‐L1 expression in tumors within 24 h. ^99m^Tc‐HYNIC‐αPD‐L1 is superior for single marker imaging due to better clinical availability, accessibility, and lower toxicity, while ^125^I‐αPD‐L1 is preferable for double marker imaging when used with ^99m^Tc‐HYNIC‐αLAG‐3. To date, several nuclear imaging tracers targeting LAG‐3 have been designed to enable real‐time visualization in vivo. For instance, PET scanning of ^68^Ga‐NOTA‐C25 revealed that LAG‐3 expression and LLC tumor uptake were both significantly increased after treated by a stimulator of interferon genes agonist or combined with PD‐1 inhibitors.^[^ ^13^ ^]^ In a preliminary clinical study, ^89^Zr‐BI 754 111 demonstrated specific binding to LAG‐3 in a limited cohort of cancer patients, with tumor uptake correlating with immune cell‐derived RNA signatures.^[^ ^14^ ^]^ However, the relationship between tracer tumor uptake and IHC expression levels of LAG‐3 has not been fully illustrated in these studies. In our study, the LAG‐3 and PD‐L1 probes demonstrated statistically significant linear correlations between tumor uptake and their respective levels on IHC. These findings indicate that it is feasible to noninvasively quantify LAG‐3 and PD‐L1 through SPECT/CT imaging, which holds great potential for guiding immunotherapy.

Both LAG‐3 and PD‐L1 are critical immune checkpoints, and the integration of LAG‐3 with PD‐L1 enhances the predictive accuracy for immunotherapy efficacy and prognosis. For instance, our previous study demonstrated that combining LAG‐3 with PD‐L1 can identify resected NSCLC patients with either the best prognosis (both negative) or the worst prognosis (both positive) (recurrence‐free survival: 2.1 vs 0.7 years; *p* = 0.007).^[^ ^6^ ^]^ Moreover, a significantly extended PFS was observed in lung cancer patients with low LAG‐3 and high PD‐L1 levels receiving PD‐1 inhibitors, compared to those with high LAG‐3 and low PD‐L1 levels (*p* = 0.005).^[^ ^4a^ ^]^ On this basis, we further performed dual index imaging with ^99m^Tc‐HYNIC‐αLAG‐3 and ^125^I‐αPD‐L1 among lung cancer models, which enabled simultaneous visualization of LAG‐3 and PD‐L1 and distinguishment of different immunotypes, accompanied by no noticeable systematic toxicity. The co‐expression of LAG‐3 with PD‐1/PD‐L1 has been documented in multiple cancers, including melanoma, lymphoma, germ cell tumors,^[^ ^4b^ ^]^ breast cancers,^[^ ^15^ ^]^ and lung cancers^[^ ^6^ ^]^ among others. Nevertheless, the predominant approach for determining the selection of combining PD‐1 with LAG‐3 inhibitors continues to rely on assessing the expression levels of the single marker PD‐L1. For example, one phase 2–3 RCT (NCT0578576) was registered to compare the efficacy of dual blockades of LAG‐3 and PD‐1 vs anti‐PD‐1 monotherapy in advanced NSCLC patients with PD‐L1 ≥ 50%. Indeed, LAG‐3 expression significantly impacts the effectiveness of dual inhibiting LAG‐3 and PD‐L1. In a large‐scale phase 2–3 RCT (NCT03470922), nivolumab plus relatlimab showed remarkably extended PFS compared to nivolumab monotherapy among melanoma patients with LAG‐3 ≥ 1% (12.6 vs 4.8 months).^[^ ^5^ ^]^ Consequently, simultaneously quantifying the expression levels of immunotherapy markers LAG‐3 and PD‐L1 is of great importance to select immunotherapy regimens. To our best knowledge, this is the first report to simultaneously visualize PD‐L1 and LAG‐3, enabling noninvasive immunotyping while shedding new light on predicting immunotherapy efficacy and guiding the choice of anti‐PD‐1 ± LAG‐3 inhibitors.

Furthermore, we observed increased tumor uptake of ^99m^Tc‐HYNIC‐αLAG‐3 probe and LAG‐3 expression on IHC in LLC‐bearing LAG‐3 humanized mice with primary resistance to immunotherapy. It indicates that LAG‐3 upregulation may be a resistance mechanism of PD‐L1 inhibitors. Similarly, LAG‐3 upregulation due to non‐cleavable LAG‐3 mutant was reported to be associated with resistance to PD‐1 blockade.^[^ ^8a^ ^]^ In short, LAG‐3 SPECT imaging provides a safe and feasible approach for monitoring immunotherapy resistance, which is crucial for guiding the intervention timing of LAG‐3‐targeting therapeutic agents.

## 4 Conclusion

SPECT/CT imaging of lung cancers with ^99m^Tc‐HYNIC‐αLAG‐3 and ^99m^Tc‐HYNIC‐αPD‐L1 probes represents an effective method for noninvasively visualizing human LAG‐3 and PD‐L1, as well as immunotherapy resistance monitoring. Additionally, a noteworthy aspect of this study is the pioneering demonstration of simultaneous imaging of LAG‐3 and PD‐L1, a development that enables the distinction of diverse immunotypes in lung cancer models. We believe the current studies are valuable for improving immunotherapeutic efficacy by properly selecting immune checkpoint inhibitors as well as their optimal intervention timing.

## 5 Experimental Section

### Antibody Chelation and ^99m^Tc Radiolabeling

The anti‐human LAG‐3 (BMS‐986016, A2029, Selleck) and PD‐L1 antibodies (MEDI4736, A2013, Selleck) were conjugated with a 20‐fold molar excess of bifunctional chelator HYNIC‐NHS (MCE). This conjugation, performed at 4 °C and pH 8.0 overnight, facilitated the synthesis of the labeling precursors HYNIC‐αLAG‐3 and HYNIC‐αPD‐L1. Unreacted HYNIC‐NHS was removed using 30 kDa ultrafiltration centrifuges purchased from Millipore Corporation. ≈10–20 millicuries (mCi) of Na^99m^TcO_4_ (Shanghai Xinke) were mixed with 100 µg SnCl_2˙_2H_2_O and 10 mg sodium glucoheptonate (GH) to generate the intermediate ^99m^Tc‐GH. Every 300 µg of labeling precursors was incubated with 37 MBq of ^99m^Tc‐GH intermediates at 37 °C for 1 h in a pH 5.6 NaAc buffer. Free ^99m^Tc‐GH and free nonreduced/reduced ^99m^Tc were eliminated through the use of 30 kDa ultrafiltration centrifugal tubes (3000 × g, 10 min for 3 times). iTLC (Scan‐RAM, SR1A/0618/503) was employed using a NaI detector (PMT‐369514) to evaluate the radiolabeling efficiency, the proportion of ^99m^Tc‐radiocolloid, and the RCP. The mobile phase comprised 0.9% NaCl and 1% EDTA, respectively. The radiolabeled compound and ^99m^Tc‐colloid remained at the origin of the silica gel 60 F254 plates (Merck), while ^99m^Tc‐GH and free nonreduced/reduced ^99m^Tc migrated to the front.

### ^125^I radiolabeling

The iodogen method was applied to radiolabel αPD‐L1 with ^125^I (Shanghai Xinke). In this process, 200 µg of iodogen (Macklin) was dissolved in dichloromethane and placed in 1.5 mL centrifuge tubes, then evaporated to dryness under nitrogen. For radiolabeling, each 200 µg of antibody was mixed with 1 mCi of ^125^I and incubated at room temperature for 15 min in 0.2 m PBS at a pH of 7.5. The reaction was halted upon removal of the mixture for purification via 30 kDa ultrafiltration centrifuges.

### In Vitro and In Vivo Stability

For assessment of in vitro stability, ≈18.5 MBq of ^99m^Tc‐labeled probes were added to 500 µL of PBS and 10% FBS, then incubated at 37 °C, to evaluate the RCP by iTLC at different time points (0,2, 4, 8, 24 h; *n* = 3). ≈3.7 MBq of ^125^I‐αPD‐L1 were added to 100 µL of PBS and 10% FBS, then incubated at 37 °C, to evaluate the RCP by radio‐HPLC at different time points (0, 24, 48, 72 h; *n* = 3). For evaluation of in vivo stability, orbital venous plexus blood collection was performed at various time points (2 and 24 h for ^99m^Tc‐labeled probes; 2, 24, 48, and 72 h for ^125^I‐αPD‐L1) after probe injection and immediately subjected to centrifugation at 1500 g for 10 min at 4 °C, and the plasma supernatants were collected. Activity of the blood samples (10 µL; *n* = 3) was analyzed by radio‐HPLC (Agilent 1260 Infinity II). UV absorbance at 280 nm was measured, and the radioactivity was detected by Flowstar^2^LB 514 (Berthold Technologies). The mobile phase, a 50 mm phosphate solution with 150 mm NaCl, allowed the separation of the radiolabeled compound from free radionuclides using a Biocore SEC‐150 column (5 µm, 7.8 × 300 mm, NanoChrom) within 18 min at 1 mL min^−1^ (Figure S12, Supporting Information).

### Cell Culture and Transfection

Human LAG‐3 gene was transfected into A549 cells using the lentivirus plasmid (PGMLV‐CMV‐H_LAG3(CD223)‐PGK‐puro) provided by Genomeditech to generate LAG‐3^+^ A549 cells. Wild‐type A549 cells (human NSCLC line) and LLC cells (mice lung cancer line) were obtained from Shanghai Pulmonary Hospital and cultured in DMEM medium. NCI‐H1975 (human lung adenocarcinoma cell line) cells, purchased from Procell, were cultured in 1640 medium. Both medias contain 10% FBS and 1% penicillin‐streptomycin. All cells were incubated in CO_2_ incubators (37 °C, 5% CO_2_).

### Western Blot (WB) Analysis

The expression levels of LAG‐3 and PD‐L1 were validated using WB. Cells were lysed using RIPA buffer, and protein concentrations were determined via the BCA method. After SDS‐PAGE and membrane transfer, the films were incubated with anti‐human LAG‐3 (Clone EPR20261, Abcam) and PD‐L1 (Clone 28‐8, Abcam) antibodies at 4 °C overnight, followed by incubation with HRP‐conjugated goat anti‐rabbit secondary antibodies (A0208, Beyotime) at 4 °C for 1 h. Glyceraldehyde 3‐phosphate dehydrogenase served as an internal reference. Bands were visualized with FluorChem M imaging system (Alpha Innotech).

### Immunofluorescence Assay

To further examine the localization of LAG‐3 expression, immunofluorescence assays were conducted. ≈5×10^4^ cells were seeded in confocal dishes and cultured for 2 days. Following fixation and blocking, cells were incubated with primary antibodies (Clone EPR20261, Abcam) and Alexa Fluor 488‐conjugated goat anti‐rabbit secondary antibodies (C2037S, Beyotime), followed by DAPI staining. Confocal microscopy images were captured with an OLYMPUS FV1200 system (Japan).

### Cell Binding Assay

LAG‐3^+^ A549, H1975, and A549 cells were seeded in 24‐well dishes with 10^5^ cells per well and incubated overnight. Two µCi ^99m^Tc/^125^I radiolabeled probes, diluted in corresponding media, were added to each well and incubated for 0.5, 1, 2, and 4 h, respectively. Each time point had triplicate wells. After dual PBS washes, cells were lysed using 0.5 m NaOH. The radioactivity in both the supernatants and cell lysates was quantified utilizing LB 2111 multi‐crystal gamma counter (Berthold Technologies). The cell binding rate was calculated by comparing the radioactivity in the lysate relative to the total radioactivity in both the supernatant and lysate, expressed as a percentage per million cells.

### Construction of Lung Cancer Models

All animal experiments adhered to protocols approved by the Institutional Animal Care and Use Committee of Soochow University (No.202306A0489). To establish lung cancer xenografts for single index imaging, female nude mice (BALB/c *Nude*) aged 4–6 weeks (Vital River) were injected subcutaneously with ≈5 × 10^6^ cells (LAG‐3^+^ A549, LAG‐3^−^ A549, and H1975) in the right flank, using a 1:1 mixture of PBS and matrix gel (354248, Corning). Bilateral lung cancer models and one unilateral model (incorporating the three aforementioned human lung cancer cells) were constructed for synchronous imaging of LAG‐3 and PD‐L1 to identify various immunotypes. In vivo imaging commenced when tumor volumes reached 200–500 mm^3^.

### Initiation of Immunotherapy

A total of 1 × 10^6^ LLC cells were injected into the right lower limb of hLAG‐3 transgenic mice (Gempharmatech). When tumor volumes reached 50–100 mm^3^, LLC‐bearing hLAG‐3 mice were treated with 10 mg kg^−1^ anti‐mouse PD‐L1 antibodies (Clone 10F.9G2, Selleck) or 0.9% NaCl as a control (*n* = 3) every three days for three times. SPECT/CT imaging of ^99m^Tc‐HYNIC‐αLAG‐3 was performed at day 13 and 14 after initiation of immunotherapy.

### SPECT/CT Imaging and Data Analysis

Tumor‐bearing mice received ≈7.4 MBq (120 µg) of ^99m^Tc‐HYNIC‐αLAG‐3 or ^99m^Tc‐HYNIC‐αPD‐L1 via tail vein injection and were scanned by microSPECT/CT system (MILabs, Utrecht, the Netherlands) under isoflurane anesthesia (*n* = 3). The block groups simultaneously took administration of a 20‐fold molar excess of unlabeled αLAG‐3 or αPD‐L1 to assess the binding specificity of probes. A 1.5 mm 75‐pinhole general‐purpose collimator and standard energy windows for ^99m^Tc and ^125^I were used. SPECT imaging was acquired at 2, 4, 8, 20, and 24 h post‐injection, with corresponding scan durations of 10, 10, 15, 20, and 30 min, respectively. CT imaging followed using a “normal full” mode of 254 seconds for SPECT data attenuation correction. Tumor models receiving both ^99m^Tc‐HYNIC‐αLAG‐3 (3.6 ± 0.3 MBq) and ^125^I‐αPD‐L1 (2.2 ± 0.2 MBq) underwent additional 48 and 72‐h imaging with 10‐min SPECT scans. Prior to ^125^I imaging, mice were administered 100 µL of 1% KI once daily for two days via gavage. With PMOD software, quantitative analysis involved delineating the ROI on CT images for tumors, blood pool, lung, liver, spleen, kidney, bladder, muscle, bone, and brain. T/M and T/B signal ratios were determined to evaluate the probe's imaging properties.

### Biodistribution

All mice were euthanized after imaging was done, and major organs were dissected, including heart, lung, liver, spleen, kidney, bladder, stomach, small intestine, large intestine, muscle, bone, blood, brain, and tumors. All tissues were weighed and measured with LB 2111 multi‐crystal gamma counter (Berthold Technologies) to describe the biodistribution profiles of ^99m^Tc‐radiolabled probes at 24 h. The biodistribution pattern of ^125^I‐αPD‐L1 was acquired through ROI analyses at 24, 48, and 72 h. After attenuation correction, the percentage of injected dose per gram (%ID/g) or per cubic centimeter (%ID/cc) tissue was calculated.

### IHC Analysis

All tumor tissues were fixed with 4% paraformaldehyde, embedded, and sectioned for immunohistochemical analysis, following protocols detailed in the previously published work.^[^ ^16^ ^]^ The primary antibodies against LAG‐3 or PD‐L1 were identical to those employed in the WB analyses. For quantification, three representative fields at 40× magnification were captured, and cell counting was performed with the ImageJ software.

### In Vivo Biological Toxicity

Following SPECT/CT imaging, blood was sampled from the mice for routine blood tests, as well as liver and kidney function tests, to evaluate the biological safety of the nuclear probes. Tumor‐bearing mice not injected with nuclear probes served as the control group. Additionally, HE staining was performed on heart, liver, spleen, lung, and kidney tissues to observe the organ‐level morphological changes.

### Statistical Analysis

All continuous data were expressed as mean ± standard deviation (SD), with sample sizes of 2–5. Normally distributed data with equal variance between two and three groups were compared using unpaired *t*‐tests and one‐way ANOVA, respectively. Pearson correlation analyses were conducted to quantify the association of in vivo tumor uptake in the ROI with *ex vivo* tumor uptake and IHC expression levels. Tumor growth curves were compared using repeated measures two‐way ANOVA. All statistical analyses were performed with GraphPad Prism 8.0.2. Two‐tailed ^*^ *p* < 0.05; ^**^ *p* < 0.01; ^***^ *p* < 0.001, ^****^ *p* < 0.0001 were considered statistically significant.

## Conflict of Interest

The authors declare no conflict of interest.

## Author Contributions

LZ, YH, and JZ designed the whole project. LZ, JG, RZ, HW, XL, KX, YL, WCZ, WGZ, LY, ZC, and MG performed the experiments, developed the methodology, analyzed and interpretated the data, drafted, and revised the manuscript. All the authors have read and approved the final manuscript.

## Supporting information



Supporting Information

## Data Availability

The data that support the findings of this study are available from the corresponding author upon reasonable request.
