# Supplementary material for: Noninvasive Immunotyping and Immunotherapy Monitoring of Lung Cancers via Nuclear Imaging of LAG‐3 and PD‐L1
Source: Adv Sci (Weinh). 2024 Nov 8;12(1):2404231. doi: 10.1002/advs.202404231 (PMC11714153; doi:10.1002/advs.202404231)
Supplement: Supplementary file 1 — Supporting Information [file ADVS-12-2404231-s001.docx]

**Supporting Information**

**Noninvasive Immunotyping and Immunotherapy Monitoring of Lung Cancers *via* Nuclear Imaging of LAG-3 and PD-L1**

Lishu Zhao^1^, Jianxian Ge^2^, Ruru Zhang^2^, Hao Wang^1^, Xinyue Liu^1^, Kandi Xu^1^, Yujin Liu^1^, Wencheng Zhao^1^, Wengang Zhang^1^, Li Ye^1^, Zhimin Chen^1^, Jianfeng Zeng^2^*, Yayi He^1^*, Mingyuan Gao^2^

^1^Department of Medical Oncology, Shanghai Pulmonary Hospital, Tongji University Medical School Cancer Institute, School of Medicine, Tongji University, No 507 Zhengmin Road, Shanghai, 200433, People's Republic of China;

^2^Center for Molecular Imaging and Nuclear Medicine, State Key Laboratory of Radiation Medicine and Protection, School for Radiological and Interdisciplinary Sciences (RAD-X), Collaborative Innovation Center of Radiation Medicine of Jiangsu Higher Education Institutions, Soochow University, Suzhou, 215123, People's Republic of China.

**Figure S1**


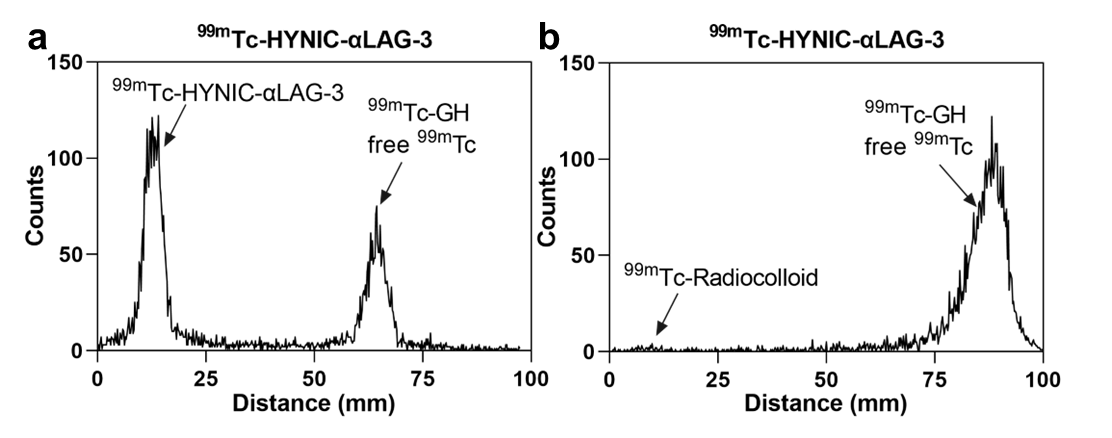


**Figure S1**. Radio TLC analyses of the labeling yield (a) and ^99m^Tc-radiocolloid (b) of ^99m^Tc-HYNIC-αLAG-3. TLC: thin layer chromatography.

**Figure S2**


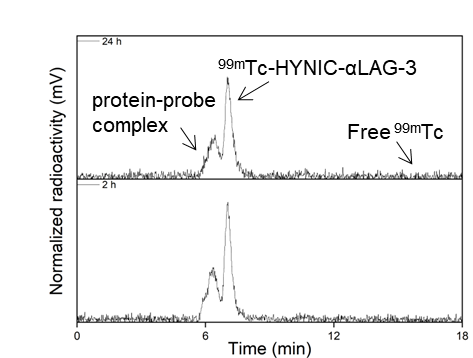


**Figure S2**. Radio-HPLC analyses of *in vivo* stability of ^99m^Tc-HYNIC-αLAG-3. HPLC: high performance liquid chromatography.

**Figure S3**


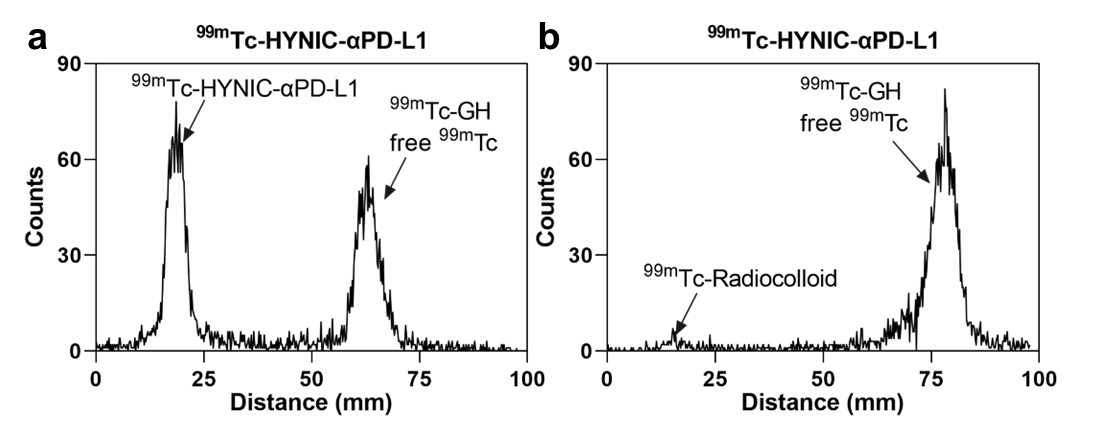


**Figure S3**. Radio TLC analyses of the labeling yield (a) and ^99m^Tc-radiocolloid (b) of 99mTc-HYNIC-αPD-L1. TLC: thin layer chromatography.

**Figure S4**


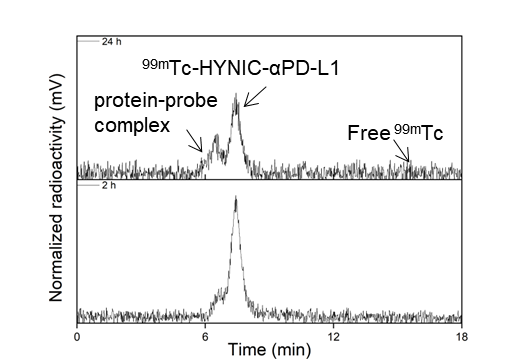


**Figure S4.** Radio-HPLC analyses of *in vivo* stability of ^99m^Tc-HYNIC-αPD-L1. HPLC: high performance liquid chromatography.

**Figure S5**


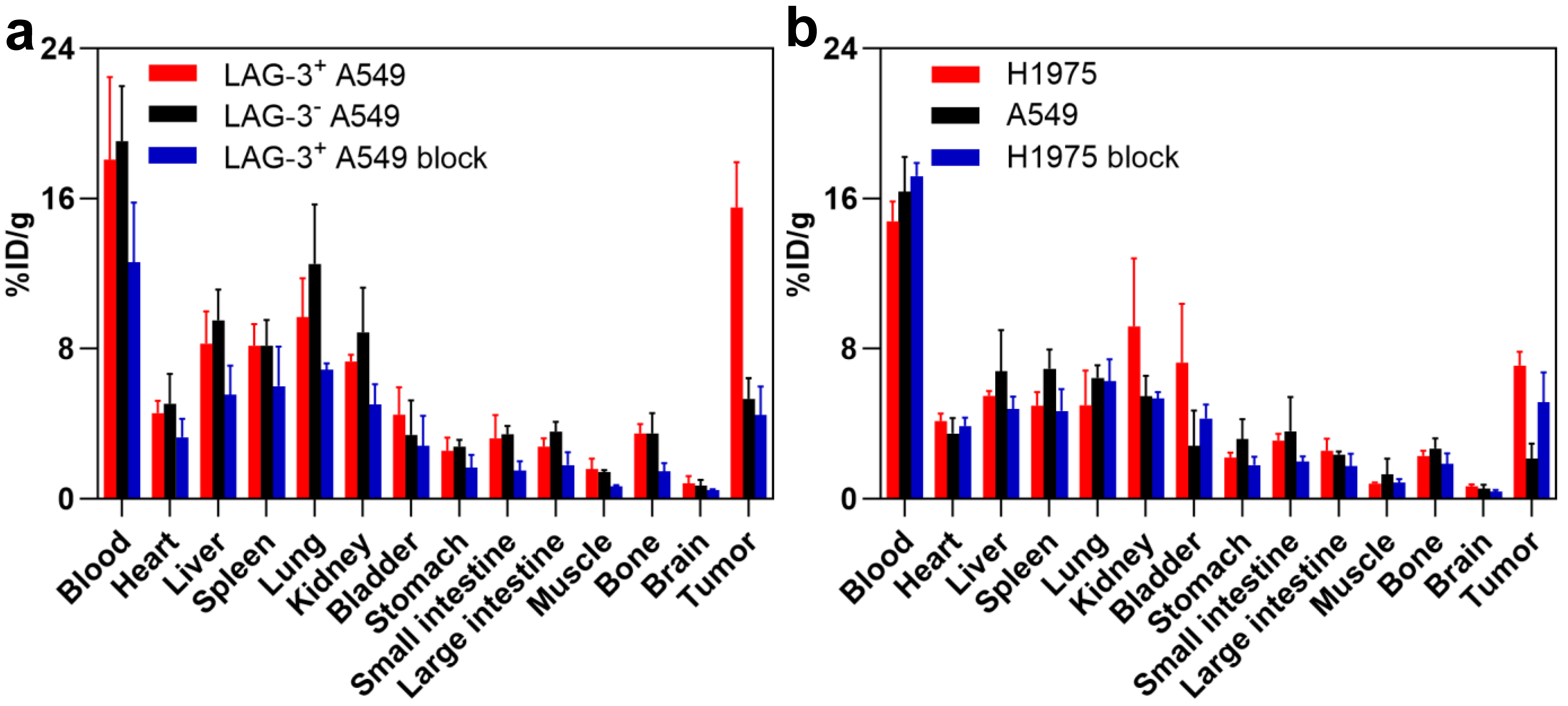


**Figure S5.** The *ex vivo* biodistribution of ^99m^Tc-HYNIC-αLAG-3 (a) and ^99m^Tc-HYNIC-αPD-L1 (b) at 24 h. Data are presented as mean ± SD, n = 3; no significance (p > 0.05) except tumor by one-way ANOVA.

**Figure S6**


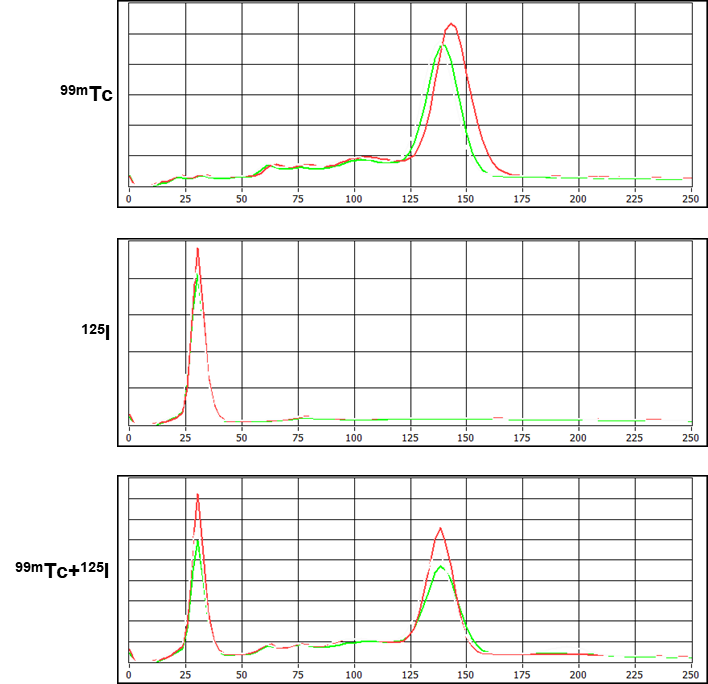


**Figure S6.** The radiation energy spectra of ^99m^Tc and ^125^I.

**Figure S7**


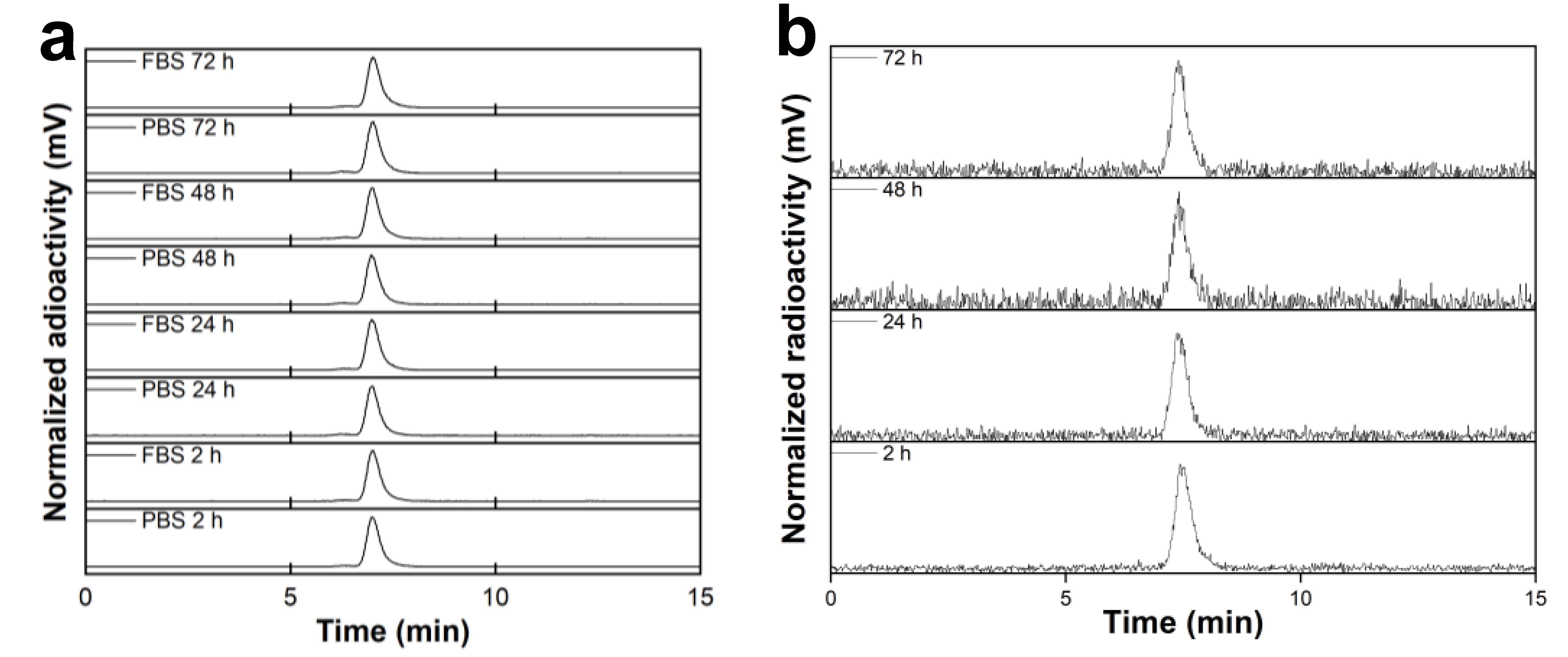


**Figure S7.** HPLC chromatograms revealed *in vitro* stability (a) of ^125^I-αPD-L1 in PBS and 10% FBS and *in vivo* stability (b) within 72 h. HPLC: high performance liquid chromatography.

**Figure S8**

**
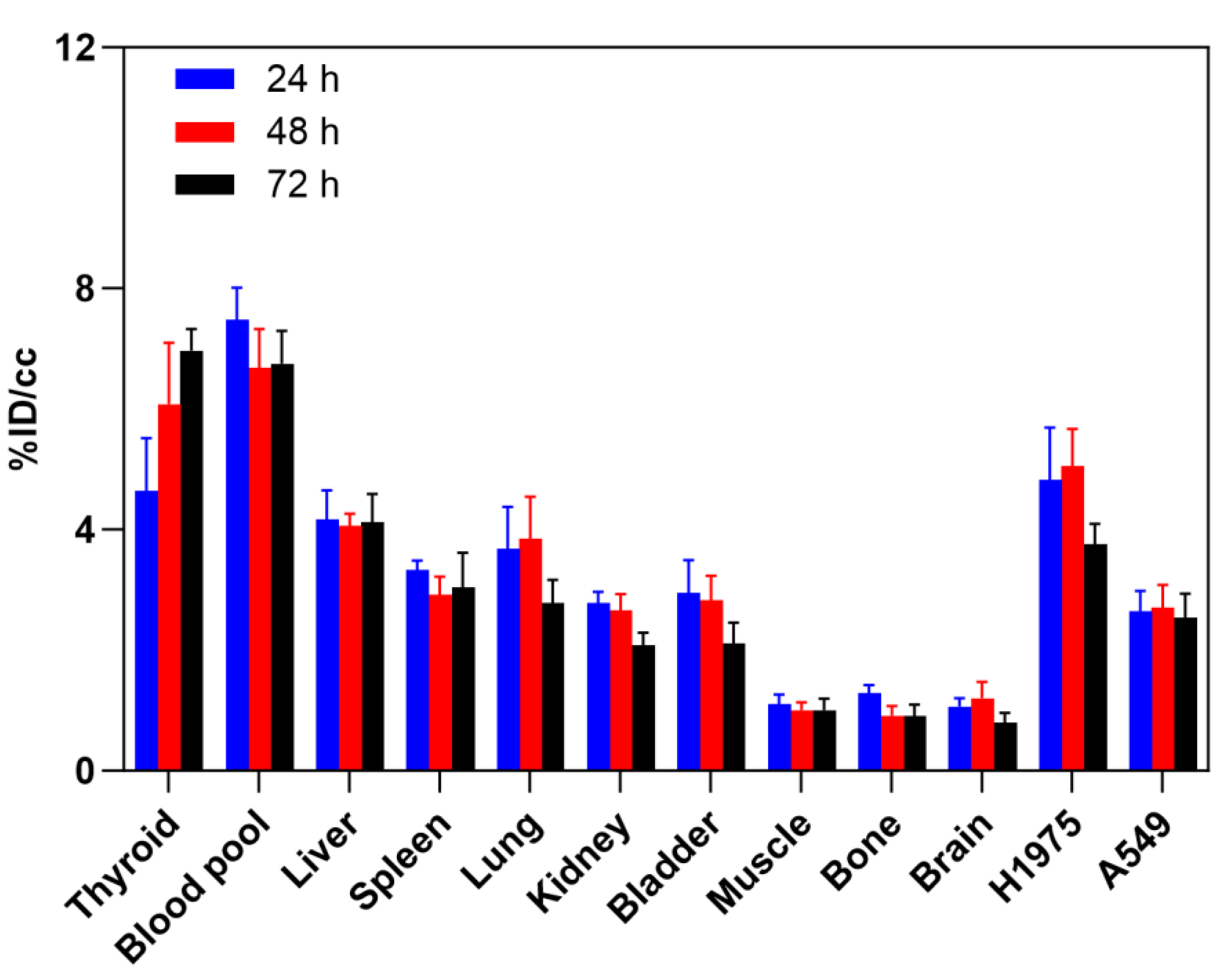
**

**Figure S8**. The biodistribution of ^125^I-αPD-L1 at 24, 48, and 72 h acquired by SPECT/CT signal. Data are presented as mean ± SD, n = 3-4.

**Figure S9**


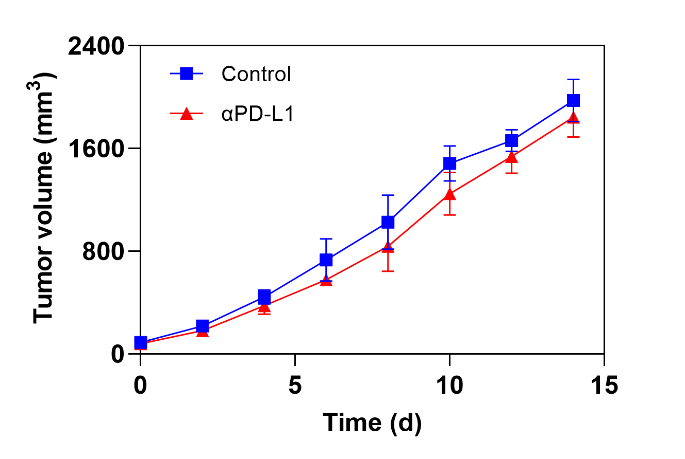


**Figure S9.** The tumor growth curve of LLC-bearing LAG-3 humanized mice between the immunotherapy group and the control group. Data are presented as mean ± SD, n = 3, no significance (p > 0.05) by repeated measures two-way ANOVA.

**Figure S10**


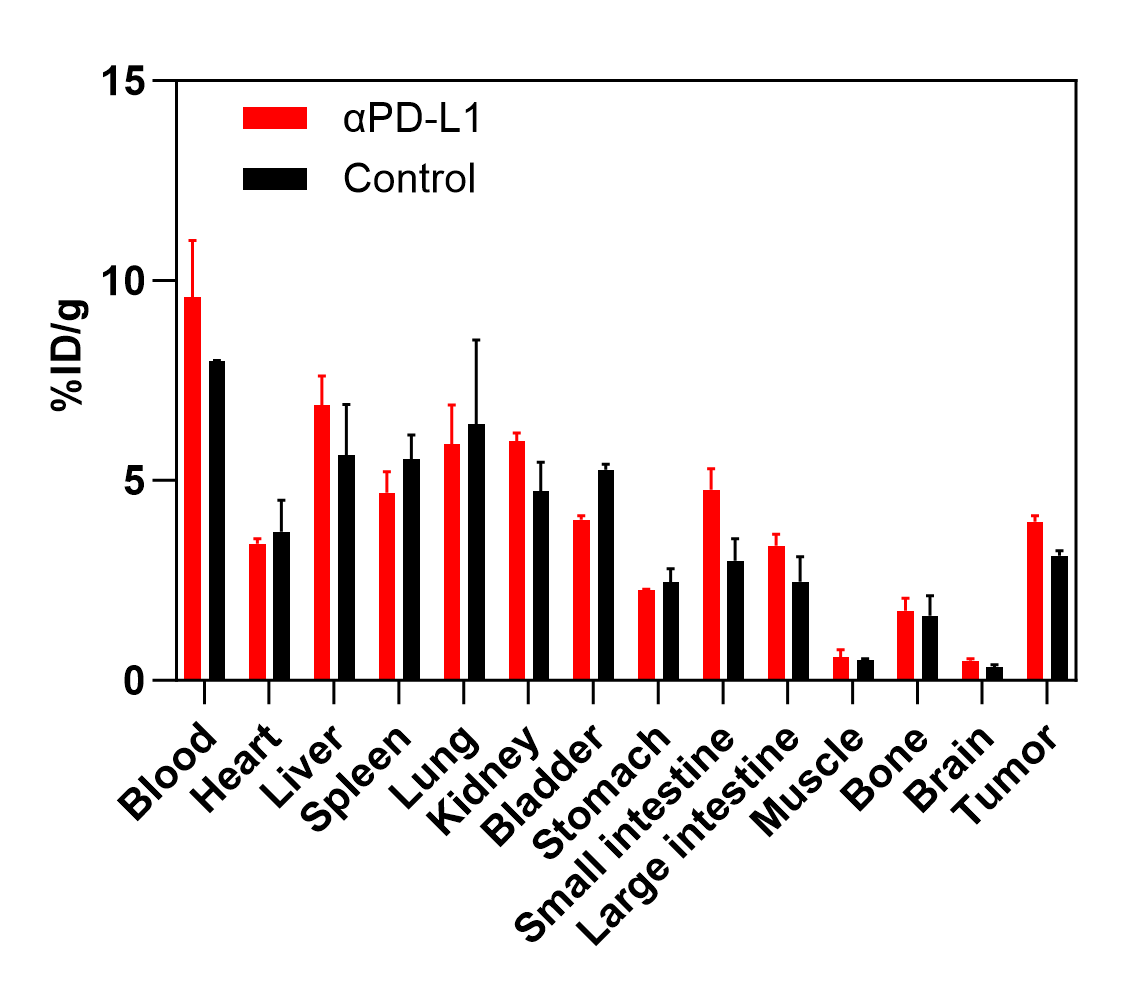


**Figure S10.** The biodistribution profile of ^99m^Tc-HYNIC-αLAG-3 between the immunotherapy group and the control group at 24 hours. Data are presented as mean ± SD, n = 3; no significance (p > 0.05) except tumor by unpaired t tests.

**Figure S11**


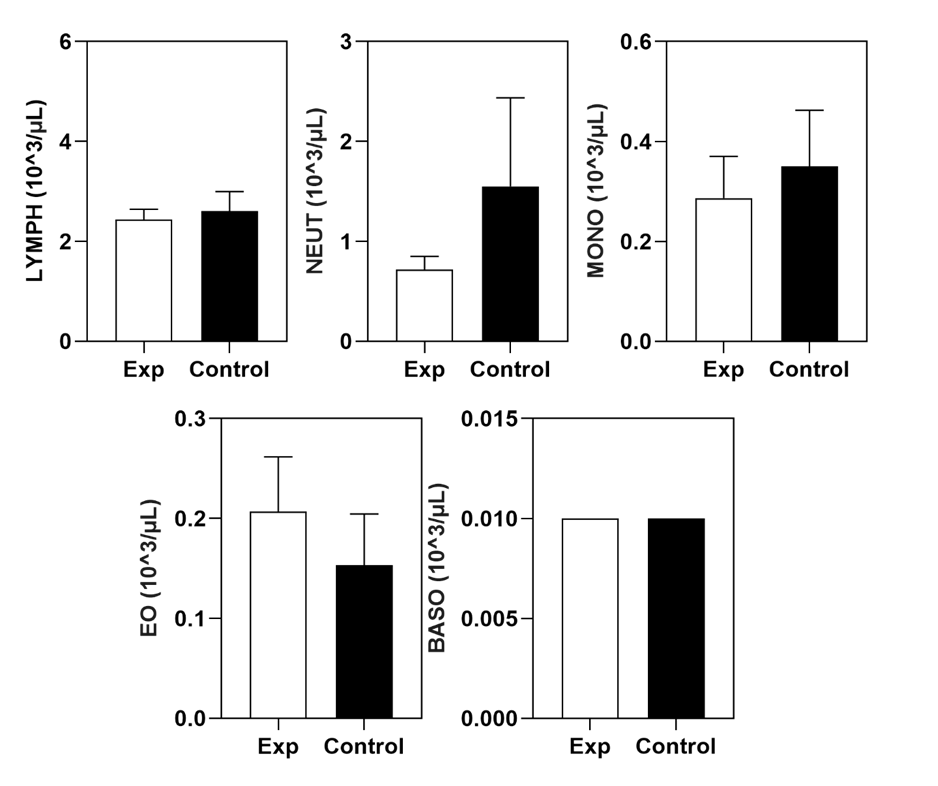


**Figure S11.** The number of NEUT, LYMPH, MONO, EO, and BASO in experiment group versus the control group. Data are presented as mean ± SD, n = 3; no significance (p > 0.05) by unpaired t tests. NEUT: neutrophils; LYMPH: lymphocytes, MONO: monocytes, EO: eosinophils, BASO: basophil granulocytes.

**Figure S12**

**
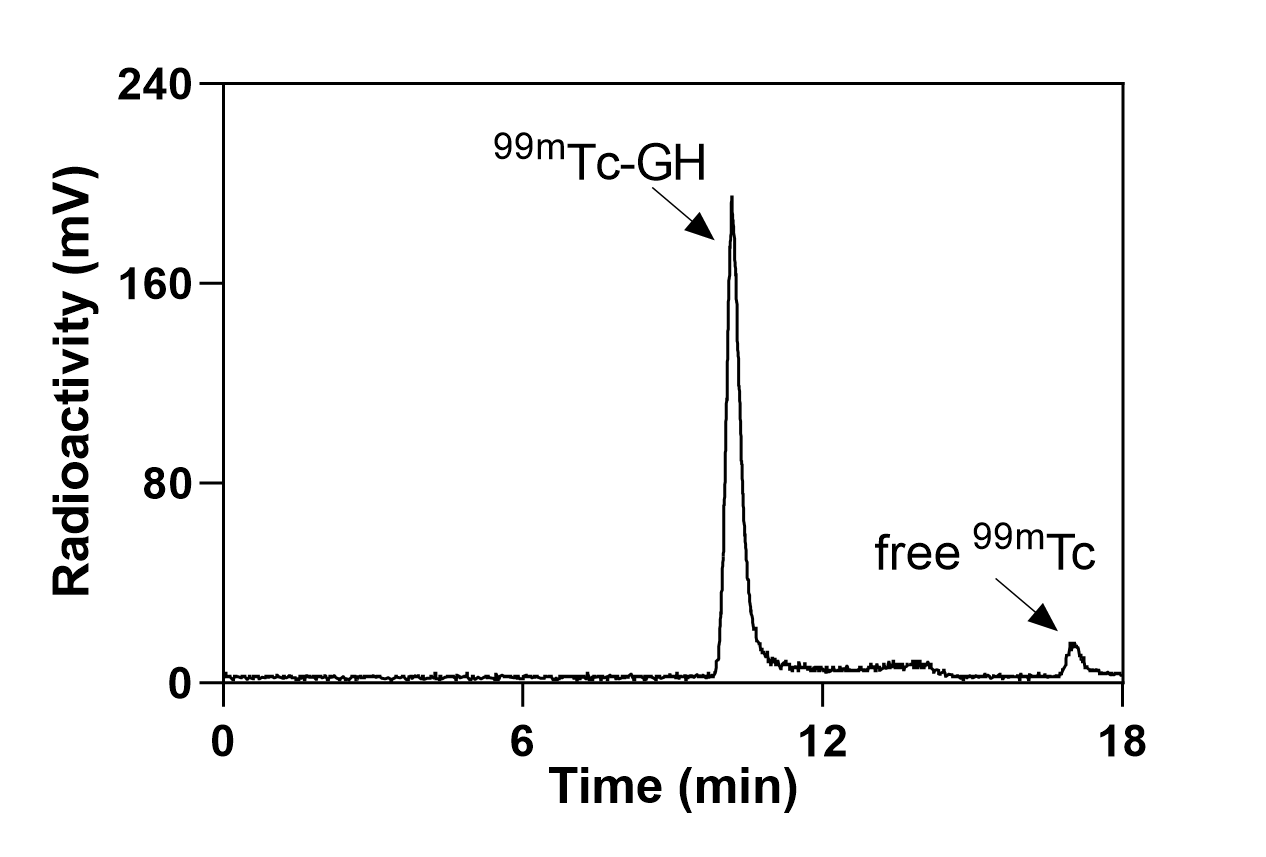
**

**Figure S12**. HPLC chromatograms of ^99m^Tc-GH and free nonreduced/reduced ^99m^Tc. HPLC: high performance liquid chromatography.
